# Management of patients with flail chest by surgical fixation using claw-type titanium plate

**DOI:** 10.1186/s13019-015-0363-1

**Published:** 2015-11-03

**Authors:** Xufeng Zhang, Zhiqiang Guo, Chuncheng Zhao, Chenyuan Xu, Zheng Wang

**Affiliations:** The Department of Thoracic Surgery, Putuo Hospital, Shanghai University of Traditional Chinese Medicine, NO.164 Lan Xi Road, Shanghai, 200062 China

**Keywords:** Flail chest, Surgical rib fixation, Mechanical ventilation, Pulmonary function, Multiple rib fracture

## Abstract

**Background:**

The goal of the study was to compare surgical rib fixation using claw-type titanium plate with conservative treatment in the management of patients with flail chest.

**Methods:**

The study retrospectively studied 23 patients suffering from flail chest injury, who admitted to our hospital from October, 2010 to February, 2014. The patients received surgical fixation by using claw-type titanium plate (surgical fixation group). A age and sex-matched cohort of 29 patients received conservative treatment and defined as conservative treatment group. Outcome variables included number of cases undergoing mechanical ventilation, ventilation time, time of hospital stay, incidence of respiratory complications, incidence of thoracic deformity and postoperative forced expiratory volume in the first second (FEV1).

**Results:**

Compared with conservative treatment group, surgical fixation group had fewer cases undergoing mechanical ventilation, shorter ventilation time, shorter hospital stay, lower incidence of respiratory complications and thoracic deformity and improved pulmonary function. Patients undergoing surgery earlier had shorter time of mechanical ventilation.

**Conclusions:**

Surgical rib fixation with claw-type titanium plate is a reliable and efficient method in the management of patients with flail chest.

## Background

Flail chest is a life-threatening injury and usually defined as the presence of three or more consecutive rib fractures in 2 or more places characterized by paradoxical motion of flailing chest wall [1]. The flail chest-related mortality ranges from 20 % to 30 % [2,3]. In addition to acute morbidities, flail chest also leads to chronic pain and disabilities.

Management of flail chest varies according to the injury severity. Conservative treatment is not sufficient to achieve a good outcome. There is growing evidence that surgical stabilization is a preferred option due to its advantages of shorter mechanical ventilation time and reduced complications caused by mechanical ventilation [4,5]. Despite its cost, surgical fixation should be recommended for treating appropriate flail chest patients [6]. Moreover, it has been reported that open reduction internal fixation using claw-type plate is an operable and reliable method for treating multiple rib fractures [7]. The study aimed to compare the surgical fixation with claw-type titanium plate with the conservative treatment in the management of patients with flail chest. This study would add weight to evidence in support of the clinical application of claw-type plate.

## Methods

### Patients

A retrospective study was performed on the patients who were diagnosed with flail chest injury [1, 8] and admitted to our hospital from October, 2010 to February, 2014. Written informed consent was received from each participant and the study protocol was approved by local ethics committee. Indications for surgical fixation included: flail chest with ≥ 3 consecutive rib fractures in ≥ 2 locations; severe paradoxical breathing. Exclusion criteria included: age <20 years or > 80 years; severe associated trauma to head or spinal cord; severe extra-thoracic injuries that was like to cause death during the follow-up; pregnancy. A total of 23 patients received surgical fixation with claw-type titanium plate and were defined surgical fixation group. Of the 23 cases, 17 were due to car accident, 4 due to fall injury and 2 due to crush injury. A age and sex matched cohort of patients with flail chest, who received conservative treatment in our hospital from October, 2010 to February, 2014, was also included by the study, and defined as conservative treatment group. In the group, 21 cases were due to car accident, 5 cases due to fall injury, 1 case due to blow injury and 2 cases due to crush injury. The patients in the conservative treatment group did not choose surgical internal fixation because of economic difficulty or personal reasons.

In accordance with a previous study [9], the flail segments were categorized into two types based on the chest radiographs: posterolateral (PL) flail segment and anterolateral (AL) flail segment. The PL flail segment referred to the posterior fractures that influenced the line of posterior rib angle; the AL flail segment referred to anterior fractures located in the area of anterior rib angle. The lung contusion was graded based on preoperative chest radiographs and computed tomographic (CT) images according to a lung contusion grading system proposed by Balci et al. [10, 11].

### Treatment method

Immediately after admission, the patients in the conservative treatment group underwent thorax stabilization using chest straps to reduce paradoxical movement of flailing chest wall. Patients without breathing difficulties were given oxygen, conventional hemostasis, pain medications, mucolytic drugs, necessary fluid resuscitation and hormone therapy in the management of lung contusion. Combined administration of antibiotics was also taken to prevent pulmonary infection. For patients with serious breathing difficulties or in coma, endotracheal intubation or tracheostomy was performed to provide mechanical ventilator support: synchronization gap mandatory ventilation (SIMV) + pressure support ventilation (PSV) + positive end-expiratory pressure (PEEP). Patients combined with shock were given fluid and blood infusion therapy. Patients combined with pneumothorax received closed thoracic drainage treatment. Treatment for multiple injuries required consultation with surgeons of relevant departments.

**Fig. 1:**
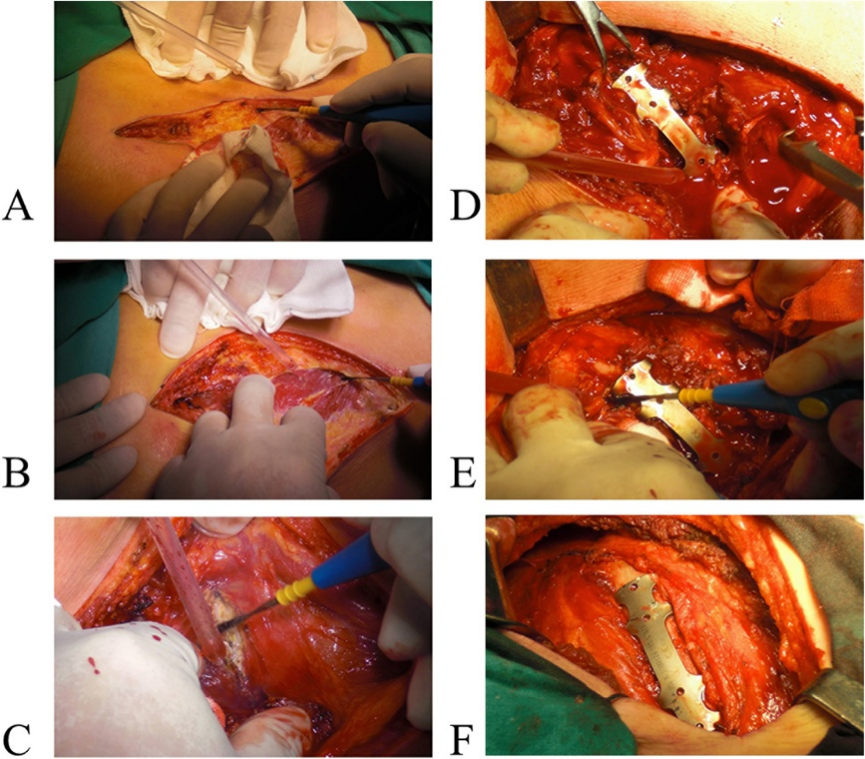
Surgical rib fixation. **a** An incision is made according to the rib fracture site, cutting the skin and subcutaneous tissue; (**b**) Then, the muscle layer is cut; (**c**) The fractured rib is exposed; (**d**) The claw-type titanium plate in appropriate size bound tightly to the fractured rib; (**e**) Steel clamp is used to adduct the claw feet of the plate so as to tightly hold the rib. (**f**) The surgery is terminated

Patients in the surgical internal fixation group also underwent the above conservative treatment. Prior to the surgical fixation, preoperative preparation was made for patients under general anesthesia and endotracheal intubation. Patients were in contralateral supine position. The position of the incision was chosen according to different rib fracture sites. Posterolateral incision was preferred in most patients. The surgery procedure was shown in Fig. 1a-e. An incision was drawn on the skin of the patient. Special care was taken to preserve the periosteum, intercostal muscle, nerves and blood vessels. Periosteum striping was avoided as much as possible. Anatomical reduction of the rib fracture edges was obtained by using reduction clamp. The fractured ribs were stabilized and fixed by using claw-type titanium plate (Changzhou Waston Medical Appliance Company, Changzhou, China) that was cut to the desired length. The plate was attached to the rib diaphysis. Steel clamp was used to adduct the claw feet of the plate as to tightly hold the rib.

Then, thoracic exploratory surgery was performed in patients requiring this surgery. A small incision was made away from the fixation site so as to prevent postoperative infection. The “coagulating” electrocautery was employed to control active bleeding. Patients with lung laceration were treated differently according to the size and depth of laceration with wedge resection as an option. After closed thoracic drainage, a layered muscular closure was performed with absorbable suture. When necessary, other surgeries were performed in patients with multiple injuries. Prior to surgical internal fixation, the patients combined with shock, subarachnoid hemorrhage or myocardial injury received other symptomatic therapy. The surgical internal fixation was implemented as soon as the patients’ condition was improved and their vital signs were stable. All surgeries were completed within 10 days following admission. Patients received routine supportive treatment postoperatively.

### Statistical analysis

SPSS 11.0 software was used for data processing in the study. The measurement data was expressed as mean ± standard deviation (x ± s). Comparisons between groups were performed using Student's *t* test or chi-square test or Fisher’s exact probability test if appropriate. *P* <0.05 represents a statistically significant difference.

## Result

### Comparison of conservative treatment and surgical fixation groups

Baseline characteristics of the patients in conservative treatment and surgical fixation groups were summarized in Table 1. There was no significant difference in age, sex, mean number of rib fractures, lung contusion severity, type of flail segment, causes and type of flail chest injury, and concomitant injuries between conservative treatment and surgical fixation groups (*P* > 0.05). Patients in the conservative treatment group received conservative therapies, while patients in the surgical fixation group received conservative therapies and surgical fixation using claw-type titanium plate. The common conservative measures of the two groups were described in Table 2. Difference of the conservative measures was insignificant between the two groups (*P* > 0.05).

**Table 1:** Baseline data of patients before treatment

	Surgical fixation group (*n* = 23)	Conservative treatment group (*n* = 29)	*P*-value
Gender (male/female)	21/8	16/7	>0.5
Age (years)	57.8 ± 12.0	59.5 ± 9.9	>0.10
Mean number of rib fracture (*n*)	7.8 ± 1.5	7.4 ± 1.7	>0.10
Grade of lung contusion	1.24	1.21	>0.5
Type of flail segment			
Anterolateral flail segment (*n*)	7	10	
Posterolateral flail segment(*n*)	16	19	
Causes			
car accident	17	21	1.000
Fall injury	4	5	
crush injury	2	2	
Blow injury	0	1	
Type of rib fracture			
bilateral	2	3	1.000
Unilateral	21	26	
Combined injury			
Spleen rupture	1	2	1.000
subarachnoid hemorrhage	1	0	0.442
Pneumothorax	12	16	0.829
Pulmonary laceration	3	0	0.160
Sternal fracture	1	0	0.442
Limb fracture	9	6	0.145
Pelvic fracture	3	5	0.976
Clavicle fracture	7	8	0.822
Myocardial injury	2	0	0.191
Mediastinal emphysema	1	2	1.000
Shock	2	3	1.000
Tracheal rupture	0	1	1.000

**Table 2:** Common conservative measures of two groups

Treatment measures	Surgical fixation group (*n* = 23)	Conservative treatment group (*n* = 29)
Chest straps (*n*)	23	29
Oxygen therapy (*n*)	23	29
Hemostasis (*n*)	23	29
Analgesia (*n*)	23	29
Mucolytic medications (*n*)	23	29
Hormone therapy (*n*)	13	17
Antibiotics (*n*)	23	29
Endotracheal intubation or tracheostomy and mechanical ventilation (*n*)	11	14
Fluid and blood infusion therapy (*n*)	2	3
Bronchoscopy for sputum suction (*n*)	10	11
Closed thoracic drainage treatment (*n*)	12	26

**Fig. 2:**
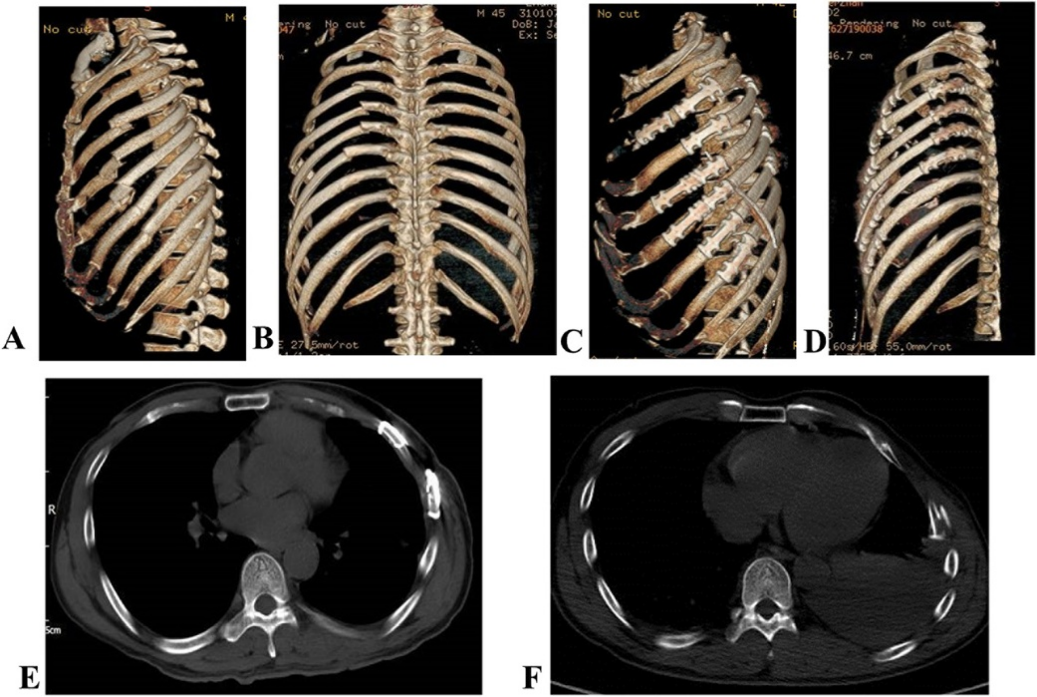
One typical case of rib fracture before and after surgical fixation. **a-b**, a 3- dimensional reconstruction CT image showing fractured rib before surgery. (**c-d**), a 3-dimensional reconstruction CT image after the surgery. (**e**), a chest CT image showing fractured rib before the surgery. (**f**), a chest CT image showing fractured rib after the surgery. Collapse of the thorax is corrected after the surgical fixation

Treatment outcomes of the patients in the two groups were compared in Table 3. It showed that surgical fixation group had significantly fewer cases taking mechanical ventilation (*P* < 0.01), shorter ventilation time and shorter ICU stay compared to the conservative treatment group (*P* < 0.01;*P* < 0.05). Moreover, incidence of respiratory complications and thoracic deformity was significantly greater in the conservative treatment group than that in the surgical fixation group (*P* < 0.005). Representative CT images of a typical case undergoing surgical fixation showed that the thoracic collapse was corrected after the surgical fixation (Fig. 2). It would help improve lung function.

**Table 3:** Treatment outcome of patients in two groups

Category	ICU stay (day)	Mechanical Ventilation (*n*/%)	Mechanical ventilator time (day)	Respiratory Complications (*n*/%)	Chest deformity (*n*/%)	Death (*n*)
Conservative Treatment (*N* = 29)	14.2 ± 6.5	24(82.76 %)	13 ± 7.6	22(75.86)	12(41.38 %)	0
Surgical Fixation (*N* = 23)	5.5 ± 6.4	11(47.83 %)	4.1 ± 6.1	7(30.43)	0	0
T or *x*^2^ value	4.826	7.113	4.567	10.731	12.372	-
*P*-value	<0.05	<0.01	<0.01	<0.005	<0.005	-

Intensive care unit, ICU

### Surgery-related complications and follow up

**Fig. 3:**
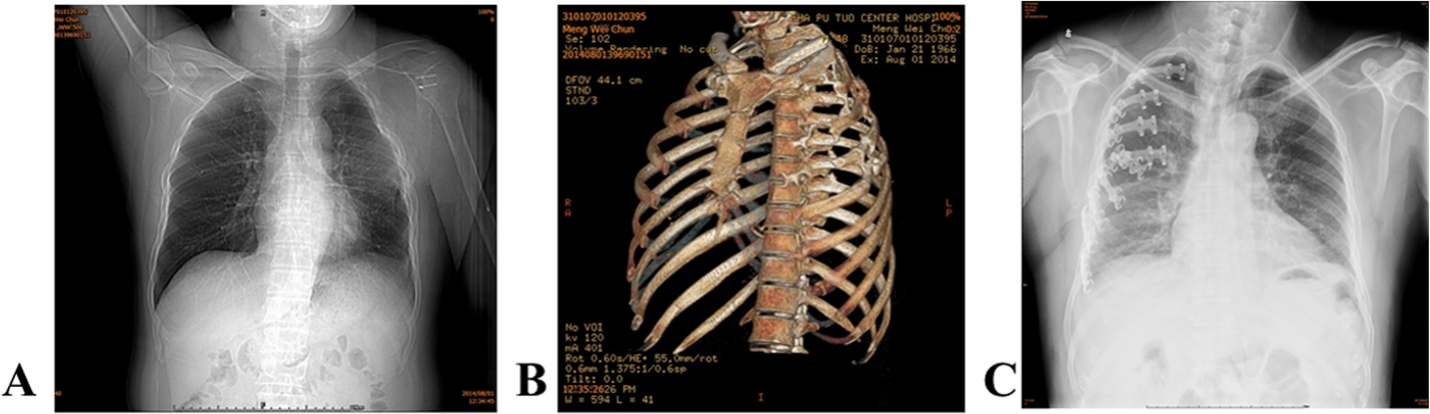
Follow-up observation. **a** a chest radiograph of the patient in surgical fixation group, who sufffers from osteomyelitis during the follow up. **b**, a chest CT image of the patient in surgical fixation group, who suffers from osteomyelitis during the follow up. **c**, a chest radiograph of a patient in surgical fixation group, who recovers well during the follow up

Postoperatively, 6 patients reported pain because of claw-compression onto neurovascular bundle. Additionally, 1 patient suffered from infection and 1 patient suffered from rejection reaction in the surgical fixation group. During follow-up, osteomyelitis occurred in the patient with infection. During the follow-up, the surgical fixation failed in the patient with infection because osteomyelitis was induced. He then received thoracoplasty (Fig. 3a-b). The surgical fixation was successful in other patients (Fig. 3c). Patients of the two groups were followed-up for 13.98 ± 3.57 months (range: 12–30 months). No death or plate fracture was reported.

### Lung function assessment

Lung function of the patients in the two groups was evaluated postoperatively (Table 4). The surgical fixation group had significantly higher postoperative forced expiratory volume in the first second (FEV1) than the conservative treatment group (*P* < 0.001), indicating a better lung function.

**Table 4:** Postoperative pulmonary function assessment

Category	Postoperative FEV1
Conservative Treatment (*N* = 29)	1.43 ± 0.06
Surgical Fixation (*N* = 23)	1.58 ± 0.08
T or *x*^2^ value	7.73
*P*-value	<0.001

### Association of time to surgery with mechanical ventilation time

In the surgical fixation group, the association between time to surgery with mechanical ventilation time was further analyzed. The median time to surgery was 4 days. Subsequently, the 23 cases in the surgical fixation group were subdivided into two subgroups: subgroup A (≤4 days, 12 cases) and subgroup B (>4 days, 11 cases). Subgroup A had significantly shorter mechanical ventilation time than subgroup B (Table 5, *P* < 0.001).

**Table 5:** Association of time to surgery with ventilation time

Time to surgery	Ventilation time (day)
≤4 days (*n* = 12)	1.8 ± 3.5
>4 days (*n* = 11)	6.6 ± 7.0
T or *x*^2^ value	2.1
*p*-value	*P* < 0.05

## Discussion

Flail chest is a consequence of multiple adjacent ribs breaking in multiple places, often accompanied by lung contusion. It is observed that fail segment moves in the direction opposite to the rest of chest wall. It has varied pathophysiological consequences, leading ultimately to respiratory and circulatory failure. The study performed a retrospective study to compare surgical fixation using claw-type plate with conservative treatment in the management of patients with flail chest. The surgical fixation group had fewer cases undergoing mechanical ventilation, shorter mechanical ventilation time, shorter ICU stay, less respiratory complications, less thoracic deformities and improved FEV1 than the conservative method group. Moreover, we found that patients with shorter time to surgery appeared to have shorter ventilation time.

The treatment of flail chest focuses on chest wall stabilization, including conservative treatment measures of packing and strapping, external fixation of fractured ribs, internal pneumatic stabilization with mechanical ventilation and surgical fixation [9]. It has been demonstrated that the long-term outcome of non-operative approach is not as optimal as expected due to persistent chest wall pain, and permanent chest wall nonunion and mal-union. Moreover, long-term observation on the outcome of patients undergoing surgical fixation for rib fractures reveals that up to 90 % of the involved patients are able to return to work with few limitation at about 2 months postoperatively [12]. The National Institute for Health and Clinical Excellence recommendations in Britain has recommended surgical rib fixation in the management of patients with severe fractures [13]. A study suggests potential indications for operative repair of fractured ribs, such as flail chest, painful, movable rib fractures resistant to conservative pain management and rib fracture nonunion [14]. Another recent study regards failure of conservative management and worsening respiratory status as indications for surgical intervention [15]. In contrast, we maintained the belief that severe paradoxical breathing was also a strong indication for surgical fixation of flail chest. In comparison with internal pneumatic stabilization with mechanical ventilation, surgical fixation could immediately achieve flail chest stabilization to improve breathing.

The study showed that surgical fixation remarkably reduced the mechanical ventilation time compared to the conservative treatment (4.1 ± 6.1 *vs* 13 ± 7.6). Surgical fixation could remarkably reduce the ventilation time, leading to reduced incidence of respiratory complication and fail chest related mortality [16]. It was similar to previous studies reporting that the ventilation time approximately ranges from 3 to 5 days [15–19]. Nevertheless, it has been reported that the ventilator support time is 6.5 ± 7.0 days for patients receiving operative chest wall stabilization [20]. The incidence of postoperative respiratory complications was 30.43 % in the present study. Previous data shows that the incidence of chest-associated pneumonia is approximate 20 % [16, 18, 21]. Varied flail chest severity and surgical device might be possible causes of these inconsistent results. Different sample size might be another reason. This study showed that surgical fixation using claw-type plate could decrease the incidence of respiratory complications and thoracic deformities compared to conservative treatment. These observations were evidences supporting the superiority of surgical fixation over conservative treatment.

Increasing studies point out that patients following conservative treatment are at high risk of developing chest deformity and atelectasis, resulting in working disabilities [22, 23]. They might have persistent chest wall pain and breathing difficulties. In the present study, chest deformity occurred in 12 of 23 (41.38 %) cases of the conservative treatment group, but was not observed in the surgical fixation group, suggesting the potent therapeutic efficacy of the surgery for preventing rib cage distortion. Similarly, Granetzny et al. has reported that the chest formality only occurs in one patient with surgical fixation [23].Besides, the patients following surgical fixation displayed a better pulmonary function than those receiving conservative treatment. Likewise, it has been reported that surgical fixation achieves better outcome of pulmonary function recovery [23].

Furthermore, the study also found that in the surgical fixation group, the patients who received surgery in 4 days after injury were weaned from mechanical ventilation earlier than those who received surgery after 4 days. Similarly, Althausen et al. has reported that the days on ventilator was positively correlated with the time to operation (r = 0.477) in patients undergoing surgical stabilization of flail chest [24]. It indicated that surgical fixation should be performed as soon as possible. Flail chest injury might be aggravated if it is not treated timely. Once callus is formed, it is difficult to fix the flail chest by using claw-type plate. Thus, the surgery should be performed in 10 days after admission.

The claw-type titanium plate employed in the study was characterized with good ductility and strong corrosion resistance. Because of these desirable properties, the claw-type plate form could be adjusted according to the injured rib morphology. The plate bound tightly to the fractured rib with four pairs of claws firmly grasping the rib. The surgical fixation caused complications. In the present study, 6 of 23 patients reported postoperative pain due to compression on blood vessels and nerves. Infection and rejection reaction occurred in 1 patient, respectively. The patient with infection occurred osteomyelitis during the follow-up. The surgical fixation failed in the patient who then received thoracoplasty. Besides, comminuted fractures, fractures adjacent to transverse processes of spine or the scapula and rib cartilage fracture were also possible causes of fixation failure of claw-type plate. Additionally, the chest wall adaption might be decreased by application of too many bone plate. What is worse, lung infection might be induced and lung function might be injured in a long term [7].

Collectively, this method was effective and minimally invasive without need for application of drilling or wires or second fixation using kirschner pin. If the patient does not exhibit indications of thoracic exploratory surgery, rib fixation could be performed outside the pleura. For patients requiring thoracic exploratory surgery, rib fixation should be performed prior to the thoracic exploratory surgery for which a small incision should be made away from the fixation site so as to prevent postoperative infection. Besides, a drainage tube was placed routinely following the surgery. The study had weaknesses. It was a retrospective study with a small sample size. Prospective randomized comparative study enrolling a large number of patients should be performed to verify and extend the results of the study.

## Conclusion

Surgical rib fixation using claw-type titanium plate confers benefits as evidenced by shorter ICU stay, shorter ventilation time, lower incidence of respiratory complications and chest deformity, and improved pulmonary function in comparison with conservative treatment in the management of patients with flail chest. Patients undergoing surgery earlier have shorter ventilation time. The study suggests that surgical rib fixation with claw-type titanium plate is a operable and effective method.

## Abbreviations

(CT): computed tomographic
(FEV1): forced expiratory volume in the first second
(PEEP): positive end-expiratory pressure
(PSV): pressure support ventilation
(SIMV): synchronization gap mandatory ventilation
(VALI): ventilator associated lung injury
(VAP): ventilator associated pneumonia
